# Safety and Sublethal Effects of Acaricides on *Stethorus punctillum*, a Neglected Key Natural Enemy of Phytophagous Mites

**DOI:** 10.3390/toxics13050346

**Published:** 2025-04-26

**Authors:** Huan Guo, Dawei Zhang, Haoyu Wang, Xiaoling He, Senshan Wang, Yanhui Lu

**Affiliations:** 1 College of Plant Protection, Gansu Agricultural University, Lanzhou 730070, China; 19996255238@163.com (H.G.); wanghaoyu00329@126.com (H.W.); 2 National Key Laboratory for Integrated Management of Crop Diseases and Pests, Institute of Plant Protection, Chinese Academy of Agricultural Sciences, Beijing 100193, China; xnzbzdw@126.com (D.Z.); 17797692934@163.com (X.H.); 3 Institute of Plant Protection, Gansu Academy of Agricultural Sciences, Lanzhou 730070, China

**Keywords:** acaricides, *S. punctillum*, toxicity, safety assessment, sublethal effect

## Abstract

*Stethorus punctillum* Weise, a predatory beetle attacking phytophagous mites in northwest China, remains underutilized for biological control. Current over-reliance on synthetic acaricides necessitates evaluation of their non-target effects on this predator, particularly their safety and sublethal impacts. Here, we assessed the acute toxicity of four acaricides to *S. punctillum* in laboratory bioassays and then focused on sublethal impacts of abamectin on adult predation efficiency and lifespan. Based on the LC_50_ values, the acute toxicities of the four acaricides tested against *S. punctillum* larvae and adults both ranked as follows (from greatest to least): abamectin > pyridaben > spirotetramat > petroleum oil. All acaricides exhibited selective toxicity (STR: 2.16–182.49) with moderate to low risk (SF: 0.46–8.71). Notably, petroleum oil, despite showing the lowest acute toxicity to *S. punctillum*, posed the highest risk to larvae (SF: 0.46–0.77). Abamectin exposures at LC_20_ or LC_50_ significantly compromised *S. punctillum* adults, prolonging prey handling time (females: 33–100%; males: 40%), reducing maximum daily predation (females: 25–50%; males: 29%), and shortening adult lifespan (females: 2.34–3.17 days; males: 3.95–5.08 days). This study assessed the safety of four commonly used acaricides for *S. punctillum*, revealing abamectin-induced impairments to key biological traits. Our findings offer critical insights for risk-aware acaricide selection and integrated spider mite management strategies in agroecosystems in northwest China.

## 1. Introduction

Agricultural intensification has expanded in northwestern China over the past several decades [1]. The global intensification of agricultural practices has emerged as a primary driver of escalating ecological degradation, precipitating declines in ecosystem service provisioning, accelerating biodiversity loss, and undermining the biocontrol capacity of predatory arthropod populations [2,3,4]. These adverse effects further entrench chemical pesticide dependence of pest management [5], wherein control failures stimulate increased chemical use. Spider mites (Acari: Tetranychidae) are destructive agricultural pests worldwide [6], of which the two-spotted spider mite, *Tetranychus urticae* Koch, is the most damaging spider mite in northwestern China [7]. Chemical-dependent pest management now leads to diminishing returns, driven by escalating pesticide resistance and disrupted biocontrol services [8].

*Stethorus punctillum* Weise, a dominant natural enemy of phytophagous mites in northwest China, is an efficient predator of spider mites with a significant potential for use as a biological control agent [9]. However, its cryptic morphology and thanatosis behavior makes it easily overlooked in agricultural fields, leading to the unintended loss of its biocontrol value due to the indiscriminate use of chemical agents. In the context of conservation biological control, modifying habitats and use of less damaging pesticides can significantly mitigate the adverse impacts of pesticides on natural enemies [10].

Pesticides can have lethal and sublethal effects on arthropods under field conditions [11], with the latter often occurring through exposure to sublethal concentrations of chemicals produced by degradation of residues over time [12,13]. Low concentrations of pesticides, including acaricides, may affect the longevity, life history parameters, and predation capacity of exposed natural enemies [14,15]. Current research on mite biological control prioritizes toxicological assessments of impacts on predatory mites, for both acaricide safety [16] and sublethal impacts [17,18]. There are relatively few studies on *S. punctillum* or other insects that feed on mites, and existing studies of *S. punctillum* mainly focus on the beetle’s biological characteristics [19,20], bioassays of direct toxicity of certain pesticides [21], and *S. punctillum*’s interactions with other mite predators [22]. Notably, there is a significant gap in the area of assessment of acaricides’ safety and sublethal impacts for this efficient predator.

In this study, we assessed four commonly used acaricides that have distinct modes of action for their acute toxicity against *T. urticae* and *S. punctillum*. The safety profiles of acaricides to the predator were evaluated by calculating the selective toxicity ratios (STR) and safety factors (SF). Additionally, the sublethal effects of abamectin (the most widely used insecticide-acaricide in northwest China) were quantified through predation behavior assays and longevity monitoring. This study advances the task of selecting acaricides suitable for use in integrated pest management (IPM) programs, while demonstrating how natural enemy conservation enables ecologically sustainable spider mite management.

## 2. Materials and Methods

### 2.1. Test Mites and Insects

*Tetranychus urticae* and *S. punctillum* were collected from an apple orchard at the Horticultural Farm in Dangzhai Town, Zhangye City, Gansu Province (38.84° N, 100.49° E). *Tetranychus urticae* was reared on *Phaseolus vulgaris* L. seedlings in the laboratory. After the second compound leaves had expanded, plants were inoculated with spider mites and watered every 5 days. *Stethorus punctillum* was reared by providing prey (*T. urticae*) on fresh, infested bean leaves, which were replaced daily. Rearings were done in an artificial climate chamber (26 ± 1 °C, relative humidity 50 ± 10%, and photoperiod 16:8 light/dark). Adult mites, 1-day-old adults and third instar larvae of *S. punctillum* were selected for subsequent experiments.

### 2.2. Acaricides and Reagents

For testing, we used technical acaricides as follows: 95% abamectin and 97% spirotetramat (from the Hebei Weiyuan Bio-Chemical Co., Ltd., Shijiazhuang, China), 95% pyridaben (from the Jiangsu KWIN Group Co., Ltd., Yancheng, China), and 99% petroleum oil emulsion (from the Shandong Lukang Bio-Pesticide Co., Ltd., Dezhou, China). Dimethyl sulfoxide (analytically pure, Sinopharm Group Chemical Reagent Co., Ltd., Shanghai, China) was used to dissolve the above-mentioned technical acaricides and dilute them into a 1% stock solution.

### 2.3. Determination of Acaricides Acute Toxicity and Safety Assessment

The stock solution was diluted with distilled water to obtain various concentrations for acute toxicity bioassays, and distilled water containing 1% dimethyl sulfoxide was used as the control (Table 1).

The acute toxicity bioassay for *T. urticae* was based on the leaf-dipping method [23]. Into Petri dishes (90 mm dia × 15 mm h) filled with 1% agar, we placed fresh bean leaves (*P. vulgaris*) that had been immersed in a test acaricide solution for 5 s and then air-dried. One leaf per Petri dish was placed bottom side up on the agar, and 30 *T. urticae* adults of uniform size were placed on the leaf. The open Petri dish was then sealed and closed with a sheet of parafilm that had microperforations for ventilation. Eggs laid by the mites were removed daily, and the number of living mites was recorded 24 h and 48 h after the start of the assay.

The acute toxicity bioassay with *S. punctillum* was based on contact with a surface residue in a test tube (80 mm h × 18 mm dia) [24], using either 1-day-old adults or third instar larvae. To create the test arena, we added 3 mL of one concentration of a particular pesticide into a test tube, which was then mechanically rolled while the pesticide air-dried at room temperature and without forced ventilation. We then introduced 10 well-fed *S. punctillum* adults or larvae into each tube, which was then sealed with a cotton plug and placed horizontally on a laboratory bench. Three hours later, test insects were transferred into small, clean polyethylene cups (25 mL), whose lids had been pricked with a needle to provide 15–20 microperforations for ventilation. Live mites were added to cups daily in excess as food for predators, and the survival of the test insects was recorded after 24, 48, and 72 h. Each treatment was replicated 3 times. Replicates were considered invalid if the mortality rate of their control group exceeded 10%.

### 2.4. Lethal and Sublethal Effects of Abamectin on Predation Ability of S. punctillum

In the laboratory, 1-day-old male and female adults of *S. punctillum* were exposed to LC_20_ and LC_50_ concentrations of abamectin using the same test tube contact assay described above. The surviving individuals after 36 h were placed individually into Petri dishes and starved for 6 h. Fresh leaves of *P. vulgaris* infested with *T. urticae* (with leaf petioles wrapped with wet cotton balls) were placed into the Petri dishes, which were then sealed with parafilm with microperforations. The densities of *T. urticae* tested were 50, 100, 150, or 200 individuals per dish. After 24 h, the numbers of surviving *T. urticae* were counted. One Petri dish with one adult predator as a replicate and each treatment was replicated 5 times, and the control was treated with distilled water containing 1% dimethyl sulfoxide. A total of 60 female adults and 60 male adults were tested in the predation bioassays.

### 2.5. Lethal and Sublethal Effects of Abamectin on Longevity of S. punctillum

The setup for the treatment and selection of ladybugs was the same as in the above section. The surviving individuals were paired after 48 h, and then the pairs were placed in Petri dishes. Subsequently, we placed fresh leaves of *P. vulgaris* infested with *T. urticae* in the Petri dishes. Leaves had their petioles wrapped with wet cotton balls, and Petri dishes were sealed with parafilm with microperforations. Infested leaves were replaced daily, and we recorded adult beetle mortality for both sexes. Each treatment was replicated 24 times.

### 2.6. Statistical Analysis

Data were recorded with Microsoft Excel 2019. Probit analysis was used (in IBM SPSS Statistics 26) to calculate the toxicity regression equation, and the LC_20_, LC_50_, and LC_90_ of four acaricides to different stages of *S. punctillum* were calculated. Statistical analysis was performed using the agricolae package in R Studio (4.4.1). Data normality was assessed using QQ plots (performance and ggplot2 packages), and homogeneity of variances was tested with Levene’s test (car package). If assumptions were violated, appropriate transformations were applied using the bestNormalize package. Once assumptions were satisfied, mean separation was performed using the least significant difference (LSD) method, and pairwise comparisons were conducted.

The safety of acaricides against *S. punctillum* was evaluated by use of the selective toxicity ratio (STR) and a safety factor (SF). STR is the ratio of the LC_50_ value of the natural enemy divided by that of the pest; when STR > 1, the acaricide was considered selective, i.e., the acaricide is safer to the natural enemy than to the pest [25,26]. SF is the LC_50_ value of an acaricide for the natural enemy divided by the field recommended dose (obtained from the China Pesticide Information Network, http://www.chinapesticide.org.cn, (accessed on 23 February 2025); when SF > 5, the acaricide was considered low risk, 5 ≥ SF > 0.5 meant moderate risk, and 0.5 ≥ SF > 0.05 meant high risk [27].

To analyze the predation functional response data, we used Holling’s Type II disk equation: $N_{a}=aNT_{r}/(1+aT_{h}N)$. The searching efficiency of *S. punctillum* on *T. urticae* was given by $S=a/(1+aT_{h}N)$. In the above equation, *N_a_* represents the number of prey eaten; *a* is the instantaneous attack rate of the predator on the prey; *T_h_* is the time needed to handle the prey; *N* is the prey density (the density of *T. urticae*); and *T_r_* is the total time of the experiment (*T_r_* = 1 d, since the experiment was set up for 24 h) [28]. The Origin 2021 program was employed to fit the functional response equations and create the graphs.

## 3. Results

### 3.1. Acaricides Acute Toxicity and Safety Assessment

Adult mites of *T. urticae*, 1-day-old adults and third instar larvae of *S. punctillum* had different sensitivities to these four acaricides (Table 2). Based on LC_50_ values, the acaricides exhibited the following toxicity gradient to both the pest and the predator: abamectin > pyridaben > spirotetramat > petroleum oil. The LC_50_ values for *S. punctillum* were significantly higher (i.e., less toxic) than the values for the pest mite.

Pyridaben demonstrated a high selective toxicity ratio between *S. punctillum* adults and larvae relative to pest mites (STR: 142.49–182.49). At the same time, adults of *S. punctillum* were only at moderate risk (SF: 3.29–4.94) from this acaricide, while larvae were at moderate to low risk (SF: 4.21–6.32, Table 2). Abamectin and spirotetramat also showed relatively high selective toxicity ratios between *S. punctillum* and the pest mites, and both were at moderate to low risk (STR: 7.61–32.66, SF: 1.51–8.71, Table 2). However, petroleum oil, which showed the lowest toxicity to *S. punctillum*, exhibited a low selective toxicity ratio between adults and larvae relative to pest mites (STR: 2.16–3.06), with adults being at moderate risk (SF: 0.66–1.09) and larvae at moderately high risk (SF: 0.46–0.77, Table 2).

### 3.2. Lethal and Sublethal Effects of Abamectin on Predation Ability of S. punctillum Adults

Exposure to the LC_20_ concentration of abamectin, compared with the control, significantly reduced the number of mites eaten by both sexes of *S. punctillum* only at the highest prey density (males: *F*_2,12_ = 6.983, *p* = 0.020; females: *F*_2,12_ = 18.902, *p* = 0.005. Table 3). Exposure to the LC_50_ concentration, compared with the control, reduced predation by male beetles significantly at 50, 100, 150, and 200 mites per dish (*F*_2,12_ = 2.633, *p* = 0.044; *F*_2,12_ = 4.448, *p* = 0.013; *F*_2,12_ = 2.905, *p* = 0.034; *F*_2,12_ = 6.983, *p* = 0.004), while predation by female beetles significantly declined only at 100, 150, and 200 mites per dish (*F*_2,12_ = 2.796, *p* = 0.049; *F*_2,12_ = 3.984, *p* = 0.017; *F*_2,12_ = 18.902, *p* < 0.001. Table 3).

**Figure 1:**
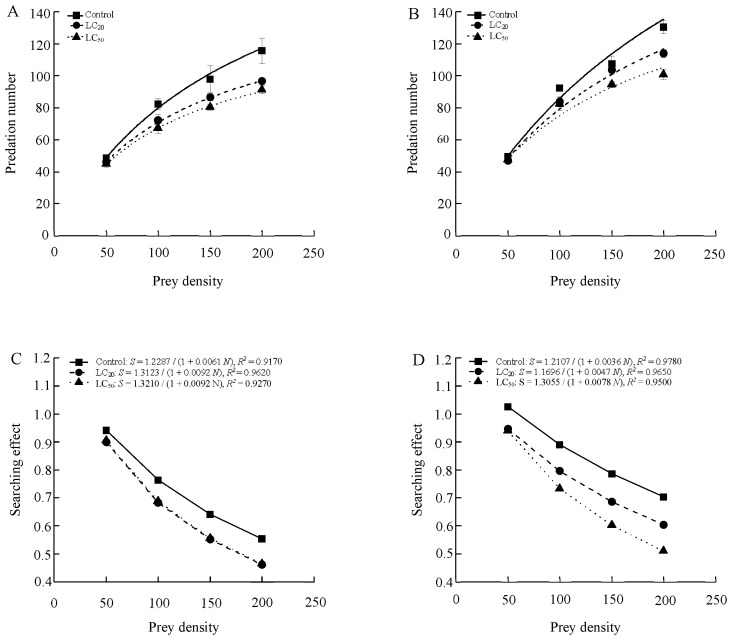
The functional response (Type II) curves and the searching effects of *S. punctillum* adults for 1-day-old males (**A**,**C**) and females (**B**,**D**).

The functional responses of 1-day-old male and female adults of *S. punctillum* to two-spotted spider mite after being exposed to abamectin both conformed to the Holling-II model (Table 4 and Figure 1). Predation increased (number of prey eaten per predator per day) with prey density before stabilizing across all treatments, and the searching effects for males and females both declined with increased prey density. Compared to the control, exposure of beetles to abamectin residues from application rates equivalent to the LC_20_ and LC_50_ levels significantly inhibited the searching efficiency of both sexes.

### 3.3. Lethal and Sublethal Effects of Abamectin on Longevity of S. punctillum Adults

**Figure 2:**
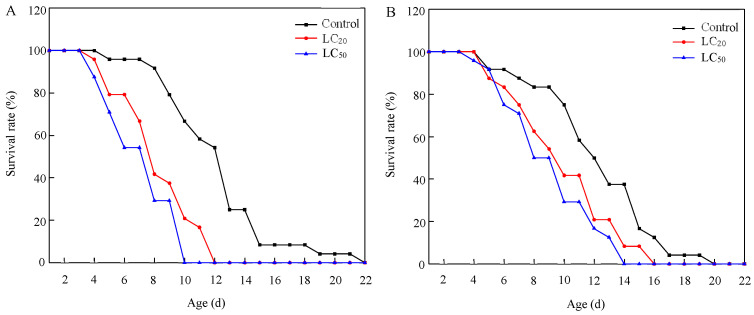
Survival curves of *S. punctillum* adults after exposure to lethal and sublethal concentrations of abamectin, for 1-day-old males (**A**) and females (**B**).

Exposure to residues from the application of abamectin significantly shortened the longevity of *S. punctillum* adults (Table 5 and Figure 2). Both LC_20_ and LC_50_ treatments significantly reduced the longevity of males and females compared to the control (males: *F*_2,69_ = 21.408, *p* < 0.001; females: *F*_2,69_ = 5.602, *p* = 0.006), and the impact on males was more pronounced.

## 4. Discussion

Agricultural intensification and expansion of acreage devoted to monoculture crops in northwest China are potentially driving biodiversity loss and escalating pesticide dependency [29]. In many crops, spider mite infestations late in the crop growth can cause substantial yield losses [30], particularly in maize fields and fruit orchards. Dense crop canopies reduce acaricide efficacy, contributing to frequent control failures and enhancing habitat and reproduction rates of spider mites in agroecosystems [31,32]. This escalation in acaricide reliance exacerbates the frequency and intensity of pesticide resistance by spider mites, inducing pest resurgence, depletion of natural enemy populations, and ecosystem destabilization [33]. Acaricide residues or patches of unevenly degraded acaricides can also have sublethal effects on non-target species, particularly the natural enemies of spider mites [34,35]. Assessing the ecotoxicological risks and sublethal impacts of field-applied acaricides on *S. punctillum* is useful for making better, more informed acaricide selection choices and conserving this predator’s ecological functions within integrated pest management (IPM) frameworks.

In this experiment, all four acaricides tested were markedly less toxic to *S. punctillum* than to the pest, *T. urticae*. The selective toxicity ratio (STR) and safety factor (SF) are metrics for assessing pesticide selectivity and informing field deployment strategies [27]. All tested acaricides had STR values > 1 for both adults and larvae of *S. punctillum*, confirming the biosafety of these products for this predator. Pyridaben exhibited pronounced selectivity toward *S. punctillum* (adults: STR = 142.49; larvae: STR = 182.49), although, notably, this contrasted with its lack of significant selectivity toward *Neoseiulus cucumeris* (Oudemans) in earlier studies [36]. This contrast likely arises from pyridaben’s mechanism of action as a mitochondrial complex I inhibitor, which selectively disrupts energy metabolism in pest mites [37], and the selectivity between *S. punctillum* and *T. urticae* may reflect divergent target-site sensitivities or enhanced detoxification pathways in *S. punctillum* [38]. SF analysis at field-recommended doses classified all four acaricides as low- or moderate-risk agents; however, petroleum oil posed a high risk to *S. punctillum* larvae (SF: 0.46–0.77), consistent with its moderate toxicity to *Halmus chalybeus* (Boisduval) [39]. This heightened risk likely stems from petroleum oil’s non-selective physical mode of action, which forms an occlusive film on the arthropod cuticle, blocking spiracles and disrupting respiration [40]. This non-selective physical action equally affects pest mites and *S. punctillum*, but the latter’s vulnerability amplified by its small body size and limited mobility. In addition, studies have shown that petroleum oil also affects the nervous system and causes dehydration and necrosis [41]. Despite its approval for use in organic agriculture, petroleum oil poses significant ecological trade-offs, and its application should be avoided during *S. punctillum*’s peak activity periods.

Abamectin is one of the most widely used insecticides and acaricides in northwest China. For that reason, it was prioritized in our study for assessment of its sublethal effects. Abamectin exhibited acute toxicity to both *T. urticae* and *S. punctillum*, likely attributable to its broad-spectrum neurotoxic action and the prolonged persistence of its residues [42]. This impact was consistent with previous research results on *T. urticae* and *Stethorus gilvifrons* (Mulsant) [43,44]. Abamectin demonstrated moderate field safety for *S. punctillum* (STR > 17.97, SF > 1.92), being less toxic to the predator than the pest mite. However, sublethal effects of abamectin via predator exposure to its residues showed a dose-dependent reduction in the predation capacity of *S. punctillum*. The maximum daily predation declined 25–50%, while adult longevity decreased by 2.34–3.17 days (females) and 3.95–5.08 days (males). These findings are similar to the sublethal impacts of abamectin on *Phytoseiulus persimilis* Athias-Henriot [45], *Phytoseius plumifer* (Canestrini & Fanzago) [46], and *Neoseiulus longispinosus* (Evans) [47]. The *S*. *punctillum*’s handling time was significantly extended, which in turn reduced its predation on pest mites. These behavioral effects of abamectin are likely due to its impact on the nervous system and endocrine homeostasis [42,48]. Despite apparent field safety, such impacts dictate that abamectin be used with caution when being integrated into pest mite control strategies.

Our research describes the safety and sublethal effects of four acaricides on *S. punctillum*. However, our results are only from indoor studies, and the number of acaricides considered was limited. Further research on acaricides is needed, and large-scale field trials should be carried out to see how environmental variables such as temperature, plant surface complexity, and acaricide degradation rates influence *S. punctillum* in natural settings.

## 5. Conclusions

In this study, we assessed the acute toxicity and safety profiles of four acaricides with distinct modes of action against *T. urticae* and *S. punctillum* under laboratory conditions. Additionally, we investigated the sublethal effects of abamectin on the predation capacity and longevity of *S. punctillum*. Although all tested acaricides demonstrated lower toxicity levels, petroleum oil posed a high risk to *S. punctillum*. Of particular concern is the substantial adverse impact of pesticide residues on both the predatory efficacy and adult longevity of this predator. The findings enhance the scientific foundation for sustainable spider mite management in northwest China.

## Figures and Tables

**Table 1:** Tested acaricides and concentrations.

Acaricides	Type	Species	Concentration Gradient (mg/L)
Abamectin	Antibiotics	Mites	0, 0.25, 0.5, 1, 2, 4
		Adults and larvae of ladybugs	0, 2, 4, 8, 20, 50
Pyridaben	Heterocyclic	Mites	0, 0.25, 0.5, 1, 2, 4
		Adults and larvae of ladybugs	0, 18.75, 37.5, 75, 150, 300
Spirotetramat	Quaternonic acid	Mites	0, 7, 14, 28, 56, 112
		Adults and larvae of ladybugs	0, 14, 28, 56, 112, 224, 448
Petroleum oil	Mineral origin	Mites, adults and larvae of ladybugs	0, 625, 1250, 2500, 5000, 10,000

**Table 2:** The acute toxicity and safety of acaricides to *S. punctillum*.

Species	Acaricides	*N*	*χ* ^2^	*df*	Slope ± SE	LC_20_ (mg/L) (95% CL)	LC_50_ (mg/L) (95% CL)	LC_90_ (mg/L) (95% CL)	Recommended Field Dose (mg/L)	STR	SF
Mites	Abamectin	540	2.424	3	2.30 ± 0.24	0.14 (0.09–0.18)	0.32 (0.26–0.38)	1.16 (0.96–1.50)	1.20–3.00	-	-
	Pyridaben	540	9.512	3	1.37 ± 0.15	0.19 (0.01–0.41)	0.77 (0.30–1.52)	6.65 (2.69–248.78)	22.23–33.35	-	-
	Spirotetramat	540	3.684	3	1.18 ± 0.15	2.87 (1.30–4.68)	14.78 (10.72–18.96)	179.44 (113.57–373.96)	44.80–74.67	-	-
	Petroleum oil	540	0.645	3	1.68 ± 1.17	446.69 (290.88–604.48)	1414.36 (1142.92–1695.85)	8180.15 (6182.31–12,177.16)	3960.00–6600.00	-	-
Adult ladybugs	Abamectin	180	2.794	3	1.25 ± 0.24	1.22 (0.36–2.26)	5.75 (3.49–8.58)	60.88 (31.49–232.14)	1.20–3.00	17.97	1.92–4.79
	Pyridaben	180	0.423	3	1.14 ± 0.26	20.07 (5.75–35.17)	109.72 (71.55–196.93)	1458.11 (555.98–16,228.88)	22.23–33.35	142.49	3.29–4.94
	Spirotetramat	180	0.018	3	0.83 ± 0.25	10.83 (0.73–23.94)	112.49 (63.13–407.53)	3973.55 (772.26–2,211,048.65)	44.80–74.67	7.61	1.51–2.51
	Petroleum oil	180	0.679	3	0.93 ± 0.26	534.40 (68.52–1087.61)	4328.38 (2581.76–10,843.12)	104,628.70 (26,965.73–8,647,436.47)	3960.00–6600.00	3.06	0.66–1.09
Larvae ladybugs	Abamectin	180	0.872	3	0.93 ± 0.22	1.31 (0.21–2.79)	10.45 (6.02–19.38)	246.62 (80.23–4507.69)	1.20–3.00	32.66	3.48–8.71
	Pyridaben	180	0.298	3	0.86 ± 0.25	14.75 (1.25–31.83)	140.52 (80.64–432.26)	4349.86 (940.86–11,239,080.53)	22.23–33.35	182.49	4.21–6.32
	Spirotetramat	180	1.365	3	0.95 ± 0.26	24.54 (3.67–48.81)	189.66 (114.35–453.64)	4269.28 (1156.41–251,385.30)	44.80–74.67	12.83	2.54–4.23
	Petroleum oil	180	0.143	3	1.11 ± 0.26	529.71 (120.90–986.00)	3050.96 (1937.22–5255.81)	43,884.84 (16,727.33–531,544.38)	3960.00–6600.00	2.16	0.46–0.77

*N* is the number of spider mites and ladybugs tested; CL is the confidence limit.

**Table 3:** Effect of lethal and sublethal concentrations of abamectin on the number of mites eaten by adults (M and F) of *S*. *punctillum*.

	Treatment	Number of Prey Eaten at Four Prey Densities (50 to 200)			
		50	100	150	200
Males	Control	48.6 ± 0.93 a	82.2 ± 3.62 a	97.8 ± 8.49 a	115.6 ± 8.02 a
	LC_20_	46 ± 0.71 ab	72 ± 3.65 ab	86.6 ± 2.11 ab	96.6 ± 1.81 b
	LC_50_	44.8 ± 1.71 b	67.4 ± 3.50 b	80.2 ± 1.93 b	91.2 ± 2.44 b
Females	Control	49.6 ± 0.24 a	92.2 ± 1.91 a	107.6 ± 4.69 a	130.4 ± 4.18 a
	LC_20_	47 ± 1.41 a	83.8 ± 3.07 ab	103.6 ± 2.56 ab	114 ± 2.51 b
	LC_50_	47.4 ± 0.93 a	82.4 ± 4.13 b	94.6 ± 2.20 b	100.8 ± 3.34 c

The data are presented as means ± SE. Data in the same column marked with different lowercase letters indicate significant differences (*p* < 0.05, LSD test).

**Table 4:** Effect of lethal and sublethal concentrations of abamectin on the functional response parameters of adults of *S. punctillum*.

	Treatment	Holling-II Disk Equation	*R* ^2^	*a*	*T_h_* (d)	*a*/*T_h_*	1/*T_h_*	*χ* ^2^	*p*
Males	Control	*N_a_* = 1.2287 *N*/(1 + 0.0061 *N*)	0.9170	1.2887	0.0050	245.74	200.00	0.131	0.988
	LC_20_	*N_a_* = 1.3123 *N*/(1 + 0.0092 *N*)	0.9620	1.3123	0.0070	187.47	142.86	0.029	0.999
	LC_50_	*N_a_* = 1.3210 *N*/(1 + 0.0092 *N*)	0.9270	1.3210	0.0070	188.71	142.86	0.036	0.998
Females	Control	*N_a_* = 1.2107 *N*/(1 + 0.0036 *N*)	0.9780	1.2107	0.0030	403.57	333.33	0.931	0.818
	LC_20_	*N_a_* = 1.1696 *N/*(1 + 0.0047 *N*)	0.9650	1.1696	0.0040	292.40	250.00	0.618	0.892
	LC_50_	*N_a_* = 1.3055 *N*/(1 + 0.0078 *N*)	0.9500	1.3055	0.0060	217.58	166.67	0.756	0.860

*a/T_h_* is the predation efficiency, where *a* is the instantaneous attack rate of the predator on the prey, *T_h_* is the time needed to handle the prey, and 1/*T_h_* is the maximum daily predation.

**Table 5:** Effect of lethal and sublethal concentrations of abamectin on the longevity of *S. punctillum*.

Treatment	Longevity of Male Adults (d)	Longevity of Female Adults (d)
Control	12.33 ± 0.73 a	12.38 ± 0.77 a
LC_20_	8.38 ± 0.51 b	10.04 ± 0.69 b
LC_50_	7.25 ± 0.46 b	9.21 ± 0.61 b

The data are presented as means ± SE. Data in the same column labeled with different lowercase letters indicate significant differences (*p* < 0.05, LSD test).

## Data Availability

The data presented in this study are available on request from the corresponding author.

## References

[1] Liang Y., Liu L. (2017). Simulating land-use change and its effect on biodiversity conservation in a watershed in northwest China. Ecosyst. Health Sustain..

[2] Wyckhuys K.A.G., Pozsgai G., Ben Fekih I., Sanchez-Garcia F.J., Elkahky M. (2024). Biodiversity loss impacts top-down regulation of insect herbivores across ecosystem boundaries. Sci. Total Environ..

[3] Wyckhuys K.A.G., Abram P.K., Barrios E., Cancino J., Collatz J., Fancelli M., Klein A.-M., Lindell C.A., Osterman J., Pinto M. (2025). Orchard systems offer low-hanging fruit for low-carbon, biodiversity-friendly farming. BioScience.

[4] Zhao Z.-H., Hui C., He D.-H., Li B.-L. (2015). Effects of agricultural intensification on ability of natural enemies to control aphids. Sci. Rep..

[5] Jaworski C.C., Thomine E., Rusch A., Lavoir A.-V., Wang S., Desneux N. (2023). Crop diversification to promote arthropod pest management: A review. Agric. Commun..

[6] Li T., Chen X.-L., Hong X.-Y. (2009). Population genetic structure of *Tetranychus urticae* and its sibling species *Tetranychus cinnabaribus* (Acari: Tetranychidae) in China as inferred from microsatellite data. Ann. Entomol. Soc. Am..

[7] Jin P.-Y., Tian L., Chen L., Hong X.-Y. (2018). Spider mites of agricultural importance in China, with focus on species composition during the last decade (2008–2017). Syst. Appl. Acarol..

[8] Bakker L., Van Der Werf W., Tittonell P., Wyckhuys K.A.G., Bianchi F.J.J.A. (2020). Neonicotinoids in global agriculture: Evidence for a new pesticide treadmill?. Ecol. Soc..

[9] Rott A.S., Ponsonby D.J. (2000). Improving the control of *Tetranychus urticae* on edible glasshouse crops using a specialist coccinellid (*Stethorus punctillum* Weise) and a generalist mite (*Amblyseius californicus* McGregor) as biocontrol agents. Biocontrol Sci. Technol..

[10] Orre Gordon G.U.S., Wratten S.D., Jonsson M., Simpson M., Hale R. (2013). ‘Attract and reward’: Combining a herbivore-induced plant volatile with floral resource supplementation-Multi-trophic level effects. Biol. Control.

[11] Desneux N., Decourtye A., Delpuech J.-M. (2007). The sublethal effects of pesticides on beneficial arthropods. Annu. Rev. Entomol..

[12] Zhang D., Dai C., Ali A., Liu Y., Pan Y., Desneux N., Lu Y. (2022). Lethal and sublethal effects of chlorantraniliprole on the migratory moths *Agrotis ipsilon* and *A. segetum*: New perspectives for pest management strategies. Pest Manag. Sci..

[13] Ullah F., Güncan A., Abbas A., Gul H., Guedes R.N., Zhang Z., Huang J., Khan K., Ghramh H., Chavarín-Gómez E. (2024). Sublethal effects of neonicotinoids on insect pests. Entomol. Gen..

[14] Zhang Q., Liu Y., Wyckhuys K., Liang H., Desneux N., Lu Y. (2020). Lethal and sublethal effects of chlorantraniliprole on *Helicoverpa armigera* adults enhance the potential for use in “attract-and-kill” control strategies. Entomol. Gen..

[15] Lutz A.L., Bertolaccini I., Scotta R.R., Curis M.C., Favaro M.A., Fernandez L.N., Sánchez D.E. (2018). Lethal and sublethal effects of chlorantraniliprole on *Spodoptera cosmioides* (Lepidoptera: Noctuidae). Pest Manag. Sci..

[16] Kuk Y.I., Kim S.S. (2018). Effects of selected insecticides on the predatory mite, *Phytoseiulus persimilis* (Acari: Phytoseiidae). J. Entomol. Sci..

[17] Sáenz-de-Cabezón Irigaray F.J., Zalom F.G., Thompson P.B. (2007). Residual toxicity of acaricides to *Galendromus occidentalis* and *Phytoseiulus persimilis* reproductive potential. Biol. Control.

[18] Ghadim Mollaloo M., Kheradmand K., Sadeghi R., Talebi A.A. (2017). Demographic analysis of sublethal effects of spiromesifen on *Neoseiulus californicus* (Acari: Phytoseiidae). Acarologia.

[19] Mehrkhou F., Fathipour Y., Talebi A.A., Kamali K., Naseri B. (2008). Population density and spatial distribution patterns of *Tetranychus urticae* (Acari, Tetranychidae) and its predator *Stethorus gilvifrons* (Coleoptera: Coccinellidae) on different agricultural crops. J. Entomol. Res. Soc..

[20] Riddick E.W., Wu Z. (2011). Lima bean-lady beetle interactions: Hooked trichomes affect survival of *Stethorus punctillum* larvae. BioControl.

[21] Shah R., Appleby M. (2019). Testing the contact and residual toxicity of selected low-risk pesticides to *Tetranychus urticae* Koch and its predators. Sarhad J. Agric..

[22] Abdellah A., Abdelaziz Z., Philipe A., Serge K., Abdelhamid E.M. (2021). Seasonal trend of *Eutetranychus orientalis* in Moroccan citrus orchards and its potential control by *Neoseiulus californicus* and *Stethorus punctillum*. Syst. Appl. Acarol..

[23] Aslam M. (2008). Evidence of field-evolved resistance to organophosphates and pyrethriods in *Chrsoperla carnea* (Neuroptera: Chrysopidae). J. Econ. Entomol..

[24] Zhang C.-X., Wang Z.-J., Li J.-J., Wang N.-M., Xue C.-B. (2022). Sublethal effects of tolfenpyrad on the development, reproduction, and predatory ability of *Chrysoperla sinica*. Ecotoxicol. Environ. Saf..

[25] Stanley J., Preetha G. (2016). Pesticide toxicity to arthropod predators: Exposure, toxicity and risk assessment methodologies. Pesticide Toxicity to Non-Target Organisms.

[26] Begna T., Ulziibayar D., Bisrat D., Jung C. (2023). Acaricidal toxicity of four essential oils, their predominant constituents, their mixtures against *Varroa* Mite, and their selectivity to honey bees (*Apis cerana* and *A. mellifera*). Insects.

[27] Yan S., Gu N., Peng M., Jiang Q., Liu E., Li Z., Yin M., Shen J., Du X., Dong M. (2022). A preparation method of nano-pesticide improves the selective toxicity toward natural enemies. Nanomaterials.

[28] Li Y., Zhang B., Zhang J., Yang N., Yang D., Zou K., Xi Y., Chen G., Zhang X. (2024). The inappropriate application of imidacloprid destroys the ability of predatory natural enemies to control pests in the food chain: A case study of the feeding behavior of *Orius similis* on *Frankliniella occidentalis*. Ecotoxicol. Environ. Saf..

[29] Suarez A., Gwozdz W. (2023). On the relation between monocultures and ecosystem services in the Global South: A review. Biol. Conserv..

[30] Jimenez L.O. (2014). Impact of Early Infestation of Two-Spotted Spider Mites (*Tetranychus urticae*) on Cotton Growth and Yield. Master’s Thesis.

[31] Liang X., Chen Q., Liu Y., Wu C., Li K., Wu M., Yao X., Qiao Y., Zhang Y., Geng Y. (2022). Identification of cassava germplasms resistant to two-spotted spider mite in China: From greenhouse large-scale screening to field validation. Front. Plant Sci..

[32] Aguilar-Fenollosa E., Ibáñez-Gual M.V., Pascual-Ruiz S., Hurtado M., Jacas J.A. (2011). Effect of ground-cover management on spider mites and their phytoseiid natural enemies in clementine mandarin orchards (I): Bottom-up regulation mechanisms. Biol. Control.

[33] Begum A., Alam S.N., Jalal Uddin M., Khan M.S., Rahman M.S. (2017). Management of pesticides: Purposes, uses, and concerns. Pesticide Residue in Foods.

[34] Müller C. (2018). Impacts of sublethal insecticide exposure on insects—Facts and knowledge gaps. Basic Appl. Ecol..

[35] Sari F. (2022). Lethal and sublethal effects of the pyrethroid insecticide tau-fluvalinate on the non-target organism *Gammarus roeseli*: A study of acute toxicity, genotoxicity and locomotor activity. Arch. Biol. Sci..

[36] Cheng S., Lin R., You Y., Lin T., Zeng Z., Yu C. (2021). Comparative sensitivity of *Neoseiulus cucumeris* and its prey *Tetranychus cinnabarinus*, after exposed to nineteen pesticides. Ecotoxicol. Environ. Saf..

[37] Jakubowska M., Dobosz R., Zawada D., Kowalska J. (2022). A review of crop protection methods against the two spotted spider mite-*Tetranychus urticae* Koch (Acari: Tetranychidae)—With special reference to alternative methods. Agriculture.

[38] Szczepaniec A., Creary S.F., Laskowski K.L., Nyrop J.P., Raupp M.J. (2011). Neonicotinoid insecticide imidacloprid causes outbreaks of spider mites on elm trees in urban landscapes. PLoS ONE.

[39] Lo P.L. (2004). Toxicity of pesticides to *Halmus chalybeus* (Coleoptera: Coccinellidae) and the effect of three fungicides on their densities in a citrus orchard. New Zeal. J. Crop Hortic. Sci..

[40] Stadler T., Buteler M. (2009). Modes of entry of petroleum distilled spray-oils into insects: A review. Bull. Insectol..

[41] Najar-Rodríguez A.J., Lavidis N.A., Mensah R.K., Choy P.T., Walter G.H. (2008). The toxicological effects of petroleum spray oils on insects-evidence for an alternative mode of action and possible new control options. Food Chem. Toxicol..

[42] Liu H., Jiang G., Zhang Y., Chen F., Li X., Yue J., Ran C., Zhao Z. (2015). Effect of six insecticides on three populations of *Bactrocera* (Tetradacus) *minax* (Diptera: Tephritidae). Curr. Pharm. Biotechnol..

[43] Tang X., Zhang Y.-J., Wu Q., Xie W., Wang S. (2014). Stage-specific expression of resistance to different acaricides in four field populations of *Tetranychus urticae* (Acari: Tetranychidae). J. Econ. Entomol..

[44] Tourani M.A., Abbasipour H. (2021). Toxicity of selected plant-derived pesticides to the citrus spider mites (Acari: Tetranychidae) and their predator, *Stethorus gilvifrons*, in the semi-field conditions. Int. J. Acarol..

[45] Monjarás-Barrera J.I., Chacón-Hernández J.C., Cerna-Chávez E., Ochoa-Fuentes Y.M., Aguirre-Uribe L.A., Landeros-Flores J. (2019). Sublethal effect of abamectin in the functional response of the predator *Phytoseiulus persimilis* (Athias-Henriot) on *Tetranychus urticae* (Koch) (Acari: Phytoseiidae, Tetranychidae). Braz. J. Biol..

[46] Hamedi N., Fathipour Y., Saber M. (2011). Sublethal effects of abamectin on the biological performance of the predatory mite, *Phytoseius plumifer* (Acari: Phytoseiidae). Exp. Appl. Acarol..

[47] Ibrahim Y.B., Yee T.S. (2000). Influence of sublethal exposure to abamectin on the biological performance of *Neoseiulus longispinosus* (Acari: Phytoseiidae). J. Econ. Entomol..

[48] El-Saber Batiha G., Alqahtani A., Ilesanmi O.B., Saati A.A., El-Mleeh A., Hetta H.F., Magdy Beshbishy A. (2020). Avermectin derivatives, pharmacokinetics, therapeutic and toxic dosages, mechanism of action, and their biological effects. Pharmaceuticals.

